# Immigrants with dementia in Swedish residential care: an exploratory study of the experiences of their family members and Nursing staff

**DOI:** 10.1186/s12877-016-0200-y

**Published:** 2016-01-16

**Authors:** Sirpa Pietilä Rosendahl, Mirkka Söderman, Monir Mazaheri

**Affiliations:** Mälardalen University, School of Health, Care and Social Welfare, Division of Caring Sciences, Eskilstuna, Sweden; Faculty of Nursing and Midwifery, Tehran University of Medical Sciences, Tehran, Iran

**Keywords:** Communication, Dementia, Family member, Group home, Nursing

## Abstract

**Background:**

Worldwide, there is a growing population of older people who develop dementia in a country other than that of their origin. When their dementia has reached an advanced stage, residential care is most often needed. People with dementia in Sweden are often cared for in group homes. For immigrants, this may mean a linguistically challenging care environment for both healthcare staff and the patients’ family members.

The aim of this study was to explore and describe the experiences of family members and professional caregivers regarding the care provided to immigrants with dementia in group homes in Sweden.

**Methods:**

An exploratory, descriptive study with a qualitative approach was chosen. In-depth semi-structured interviews were conducted with nine professional caregivers and five family members of people with dementia with Finnish, Estonian, Hungarian and Ingrian backgrounds; all were chosen purposefully. All people with dementia had lost their Swedish language skills as their second language. The data was analysed using qualitative content analysis.

**Results:**

Three main categories and seven subcategories were identified. The first main category: A new living situation comprised the subcategories: *adjusting to new living arrangements* and *expectations regarding activities and traditional food at the group home*, the second main category: Challenges in communication with the subcategories:* limited communication between the immigrant with dementia and the Swedish-speaking nursing staff* and* the consequences of linguistic misunderstandings* and *nuanced communication in a common language* and the third main category: The role of the family member at the group home with the subcategories: *a link to the healthy life story of the family member with dementia *and *an expert and interpreter for the nursing staff*.

**Conclusions:**

The family member played a crucial role in the lives of immigrants with dementia living in a group home by facilitating communication between the nursing staff and the PWD and also by making it possible for PWD to access the cultural activities they wanted and which professional caregivers were either not able to recognise as needed or could not deliver.

## Background

The number of people who, as they grow older, develop dementia in a country other than that of their origin continues to increase. This has implications for healthcare policy makers and professionals. In Europe, 9.1% (64.1 million people) have immigrant backgrounds or belong to ethnic minorities [1]. In Sweden, 21% (2,601,190) of the whole population, including 13% of people older than 65, have immigrant backgrounds [2]. The term “older immigrant” is broad and includes varying types of older immigrants with diverse motives for why and when they immigrated. Immigrants who came to Sweden in the 1950s differ from those who immigrate today in that the immigrants of the 1950s were younger and came as workers or refugees from Finland and the Baltic countries after the Second World War. In contrast, the later-life immigrants of today often come at older ages from countries outside of Europe as family-based immigrants. The term immigrant in this study refers to those who left their home country, did not speak Swedish as their first language and intended to live in Sweden permanently. The Finns came to Sweden in the 1950s to join the industrial workforce and immigrants from the Baltic countries came as refugees after the Second World War. The largest group of workers, 1.6 % per cent (*n* = 158,488), came from Finland [3]. Most Finnish immigrants learnt Swedish and became well integrated into the Swedish society; however, with age, the risk of deteriorating health increases as does the need for healthcare services. Because of this increase and today’s large waves of immigration, healthcare staff face cultural and communicative challenges from many groups. One such group includes people with cognitive decline or dementia.

Dementia is a syndrome of symptoms of cognitive decline, characterised by impaired memory function, a reduction in executive, visuospatial ability and a reduction in the ability to communicate. PWDs experience difficulties in interpreting and understanding others and have difficulties expressing themselves [4]. Bilingual immigrants with dementia lose their latest learnt language and speak their first language as the disease progresses [5, 6]. Losing their second language affects the ability of immigrants with dementia to communicate with people in their host country, including professional healthcare caregivers. In addition, the risk of negative consequences increases when neither party speaks the other’s language.

In Sweden, people who are in the initial stages of dementia live in their own homes with, depending on their family situation, a spouse or children taking responsibility for their care for as long as possible. However, as the disease progresses into its advanced stages, residential care provided by professional caregivers is necessary. Earlier research has shown that immigrants hesitate to seek formal care and do so later than native Swedes [7].

Reasons for this may be related to cultural views, that dementia is perceived as a normal part of aging and that it is the family member’s responsibility to take care of their older family members with dementia [8, 9] or that dementia is associated with a stigma and that it is shameful to have a cognitive disability [10]. International studies have also reported that a lack of knowledge about the care services available for immigrants with dementia [11, 12] or the fear of leaving a loved one with dementia in alien or foreign residential care can be reasons for seeking professional care later [13, 14]. Furthermore, studies conducted in the USA, Canada, the UK and Sweden have also reported difficulties in recruiting immigrant participants in dementia research [15].

In the past decade, there has been a change in policy regarding residential care in Sweden. The number of places in residential care has decreased as PWDs are expected to live in their own homes for as long as possible [16, 17]. This, in turn, has increased the demand on the PWDs’ family members, who have to care for their loved ones around the clock. Family member in this study implies; spouses or adult children, taking care of an older family member with dementia. This care is usually voluntary, unpaid and is generally provided until residential care is necessary [18]. However, deciding to move a PWD to a group home can be fraught with negative expectations and fears regarding the new living situation, and is not an easy decision for the family member to make.

Sweden’s healthcare system is financed by state taxes. Formal or residential care is a care service offered by municipalities and county councils at a reduced cost for the person receiving the care [18]. PWDs are usually cared for in group homes, which is based on the idea that the care environment should be as homelike as possible. The group homes are built for PWD and are usually smaller units, which have more nursing staff than an ordinary nursing home for older people. The professional caregivers are often enrolled nurses and some are specially trained to care for PWDs [17]. All residents must be diagnosed as having dementia before they can be placed in a group home. Most immigrants with dementia are placed in group homes where the staff speak Swedish. However, care for older people in Sweden has seen an increase in bilingual nursing staff, and bilingual nursing staff are sometimes matched with immigrant patients who speak the same language [5, 19, 20]. A study [21] on enrolled nurses with immigrant background in Sweden who provided care for PWD from the same background in a group home showed considerable differences in perceptions of the conscience and how to keep clear conscience compared to Swedish enrolled nurses who provided care for Swedish PWD [22–24].

A study on family members of PWD with immigrant background who still are cared at their own homes in Sweden [25] showed that family members found their care as an experience of fulfilment. They had difficulties in admitting the diagnosis of dementia for their loved one and they came to the terms with dementia when their loved one was no longer able to recognise them. Studies of immigrants with dementia in residential care in Sweden are scarce, mainly because of cultural views or a lack of knowledge regarding dementia, but also because the number of immigrants with dementia in residential care has, so far, been rather low. However, as the older immigrant population grows and ages, the risk of developing dementia increases and Swedish old age care will have more PWD from diverse ethnic origins than earlier. More knowledge is needed to provide dementia care which is multicultural. This study focuses on the perspectives of the family members and the professional caregivers of immigrants with dementia in residential care.

The aim of this study was to explore and describe the experiences of family members and professional caregivers regarding the care provided to immigrants with dementia in group homes in Sweden.

## Method

An exploratory, descriptive design with a qualitative approach was chosen as the purpose of the study was to understand and systematically describe personal experiences of the care provided to immigrants with dementia and the experiences of their family members and nursing staff. Additionally, a variety of experiences deepens the understanding of a studied phenomenon. Personal experiences are also context-bound and have to be interpreted and understood in relation to their social context [26] and, in this care context, the immigrants with dementia were cared for by professional caregivers.

### The setting

To be able to explore the experiences of both the family members and the nursing staff, the setting for this study comprised four individual group homes for people with dementia located in a middle-sized city in southern Sweden. The reason why four group homes were included was that each PWD in this study lived in a separate group home. Each group home had 10–15 PWD as residents including one or two PWD with immigrant background. Each resident had their own rooms and private furniture from their original home and they shared a living room/dining room where meals were served. The nursing care was mainly provided by enrolled nurses in all the nursing homes. The number of nursing staff members were 11 enrolled nurses working day shifts and 3 enrolled nurses for the night shifts in each group home.

### Participants

Fourteen participants were included in this study; of these, five were the family member of a PWD (2 male spouses, 1 son, 2 daughters) and nine were professional caregivers, i.e. enrolled nurses, which were recruited from the four different group homes. All enrolled nurses have vocational education in nursing care as their three years upper secondary education. The family member’s frequency of visits varied from once every other week to several times a week and over a period of six months to eight years. All the family members had cared for their loved one with dementia prior to them moving to the group home. The family members were fluent Swedish speakers. However, one of the spouses, a native Swede did not speak his wife’s native language (Hungarian), and another, a son, born in Sweden, did not speak his mother’s native language (Estonian). The immigrants with dementia came from Finland, Estonia, Hungary and Ingria and had immigrated to Sweden between 1945 and the 1950s as workers or refugees after the Second World War. All except two had spoken fluent Swedish. One immigrant with dementia did not speak any Swedish and a second had spoken enough Swedish to get by in daily life. This meant that when they lost their second language, i.e. Swedish, communication between the immigrants with dementia and their family members who spoke only Swedish was limited. The range of the native language ability among the immigrants with dementia varied; from one being very verbal to those with a fragmented or very limited language ability.

Of the enrolled nurses, five were ‘contact nurses’ for the immigrant with dementia, meaning that in comparison to the other nursing staff in the group homes, they had the main responsibility for that particular resident. This included ensuring that the PWD had suitable personal care products and that the room was clean. The contact nurses also accompanied the PWD when visiting the doctor or dentist. However, all enrolled nurses provided care for PWD with an immigrant background. All of the enrolled nurses were female, six of them only spoke Swedish and three were bilingual, speaking Hungarian, Estonian and Finnish (Table 1).

**Table 1:** Participants

Relationship to		Family	Enrolled nurses
PWD Characteristics			
Country of origin	Finland	1	1
	Estonia		1
	Hungary	1	1
	Ingria	1	0
	Sweden	2	6
Time of immigration		1945–1950	
Reason for the PWD emigrating	Work	2	
	Refugee	3	
Gender	Female	3	9
	Male	2	0
Family role	Daughter of PWD	2	
	Son of PWD	1	
	Spouse of PWD	2	
Age		40–80	30–60
Mother tongue	Swedish	2	6
	Finnish	1	1
	Hungarian	1	1
	Estonian	1	1

### Data collection

The participants were recruited using purposive sampling with the inclusion criteria that they should be a family member (spouses or adult children), who visited the immigrant with dementia regularly and professional caregivers working in the group homes for the immigrants with dementia. After initial contact and being given permission by the unit’s superintendent to conduct the study, the researcher (SPR) was introduced at the group homes and to the professional caregivers. The first author (SPR) provided the staff with oral and written information about the study. The family members were first contacted by the nurses in charge at the group homes who distributed written information about the study and invited the family members of the immigrants with dementia to participate. If they agreed to participate, they were then contacted by the first author (SPR) and given additional information about the study.

The participants chose to be interviewed in a quiet place at the group home. With the participants’ permission, the interviews were audio-recorded. The interviews lasted for approximately 45 min. A semi-structured interview guide with questions related to the purpose of this study was at hand. Examples of questions asked see Table 2.

**Table 2:** interview guide

Questions were used in interviews with enrolled nurses	• Can you describe an ordinary working day! • What is your experience of caregiving of immigrants with dementia? • How do you communicate to make yourself understood? • What cultural commonalities/differences are there between the residents? • What cultural differences are there?
Questions were used in interviews with family members	• Can you tell me why your mother/father/spouse moved to the group home? • What do you think your loved one experienced moving to a group home? • How do you think he/she has accepted their new housing arrangements? • How do you think they manage communication here, not speaking each other’s languages? • What kind of cultural commonalities/differences are there in the care of people with dementia here [in Sweden] and in your country? • How do you experience your visits here (to the group home)?

### Data analysis

The interviews were transcribed verbatim by the first author (SPR) and the data were analysed according to qualitative content analysis as described by Krippendorff [26]. As a widely used research technique, qualitative content analysis [27] has been used when studying a wide variety of subject matter. It is particularly suitable when analyzing interview data in care science research and education [26, 28]. The transcribed interview material was analysed according to the steps described below.

Firstly, all transcribed interviews were read through several times by the first (SPR) and second (MS) authors to get an overview of the content. Both authors (SPR, MS) were involved in the whole process of analysis. Secondly, content or meaning units related to the study’s aim were identified. In this context, a meaning unit consisted of words, sentences or paragraphs containing aspects that were inter-related through content or context. Thirdly, the meaning units were condensed; this meant shortening the text without reducing the core content of the meaning unit. Fourthly, the condensed meaning unit was labelled with a code summarising the content of the meaning unit. Fifthly, codes and meaning units with similar content were categorised. Finally, categories with similar content were abstracted into subcategories and, for higher levels of abstraction, into main categories [26]. The analysis of this study resulted in three main categories and seven subcategories, which are presented in the results section.

### Ethical considerations

This study was approved by the ethical committee at Linköping University, Linköping, Sweden (Dnr 03–264). After receiving ethical approval, the potential participants received oral and written information about the study, its purpose and data collection procedures, that participation was voluntary and that the participants could withdraw from the study at any time without having to give a reason. They also received information regarding the confidential storage of the data in a secure location to which only the researchers involved in the study had access. Furthermore, the participants were told that their real names and locations would be changed so that no participant could be identified. The participants signed the written informed consent.

## Results

The presentation of the results is based on the categories identified in the analysis and in relation to the purpose of the study, which was to explore and describe the experiences of family members and professional caregivers regarding the care provided to immigrants with dementia living in group homes in Sweden. For immigrants with dementia, moving to residential care meant a new living situation and was challenging in terms of adjusting and communicating in a new care environment with new routines and new relationships. For their family member, the move meant a new role (Table 3).

**Table 3:** Main/subcategories

Main categories	Subcategories
A new living situation	Adjusting to new living arrangements Expectations regarding activities and traditional food at the group home
Challenges in communication	Limited communication between the immigrant with dementia and the Swedish-speaking nursing staff The consequences of linguistic misunderstandings Nuanced communication in a common language
The role of the family member at the group home	A link to the healthy life story of the family member with dementia An expert and interpreter for the nursing staff

## A new living situation

Prior to their loved one moving to a group home, the family member, spouse or children, had either provided around-the-clock care for their family member with dementia or had closely monitored their ageing loved one’s situation through frequent visits. However, the time would come for their loved one to move to another type of housing. This would be a time for adjusting to a new living situation. The category, a new living situation included the subcategories ‘adjusting to new living arrangements’ and ‘expectation regarding activities and traditional food at the group home’.

### Adjusting to new living arrangements

One of the family, a spouse who had cared for his wife until he ended up in the emergency ward, would have gladly continued caring for his wife, but realised he could not manage any longer. He meant that a condition of caring in the home was that family members, children and relatives would help, otherwise, it was too burdensome. It was also a relief to another family member when their older family member with dementia was given a place in a care facility and that this facility was suited to PWD. Another family member was of the opinion that leaving one’s home to move to a group home was more significant than the fact that the PWD had an immigrant background and was, perhaps, going to a caregiving environment that was culturally foreign to them. Adjusting to a new living situation varied among the immigrants with dementia. Some showed signs of being homesick at the beginning and were inclined to try to run away; however, after a period of time, they accepted their new living arrangements.

(Spouse about Z) *“She is doing fine here (now). In the beginning I knew, as did her carers, that she would be homesick, and she was and when I left I sort of had to sneak out.”*

(Enrolled nurse about Z) “*I think it has gotten better in the last few days, the last week… but that’s her medication. It was terrible to see her wrenching and pulling at the door, and she was just heartbroken that she couldn’t get out, and it didn’t matter what we tried because it wouldn’t get her out of there. She put on three coats, packed her bag and stood there.”*

Not everyone found it difficult to adjust to their new living arrangements. The daughter of a Finnish PWD had had her mother staying with her on and off, but when her mother moved to the group home, she (her mother) coped with the new situation immediately.

(Daughter) “*…and she felt at home there right away, there were people there, that meant a lot, but she hadn’t realised that earlier I don’t think, so I feel it’s like she has just blossomed.”*

Although most of the family members were satisfied with the new living arrangements, they also spoke of some of their expectations regarding the care provided.

### Expectations regarding activities and traditional food at the group home

The family members observed the daily life in the group home and all of them were satisfied with the basic care that their loved ones received; however, they had noticed that opportunities for activities apart from basic care were scarce. By that they meant physical activities, like spending time outside, going for walks, going on trips and receiving occupational therapy, all of which were limited or missing. It seemed, from their point of view, that the PWDs were made to rest during the day instead of being involved in activities.

(Interviewer) *“What do you think it is like here for her?”* (Son) *“Well, what can I say, I think basically it’s perfectly fine, but I don’t think they have enough time. They are happy to put people to bed whenever. They say they have to, I think it gives them a break, they are short of staff.”*

Although activities of a more physical nature were limited, there were some that did not require much physical energy or strength. During the day and in between the meals, the PWD listened to music, watched television or looked at magazines on their own or with the nursing staff. The residents did not seem to socialise with each other. For the immigrants with dementia, it was obvious that they preferred music and television programmes that they were familiar with in their native language.

(Daughter) *“I don’t know if they interact much with each other, each lives in his or her own world, but when there’s music, I notice that she gets excited. Never Swedish music, just Finnish music and she listens to the news in Finnish, and if she’s in the day room she stays when the Finnish news is on. She likes to watch and listen to that, but she listens to Swedish programmes too sometimes when she’s in the dayroom.”*

On the other hand, the professional caregivers said that activities, apart from care, could depend on the residents’ health status on that day, in that people with severe dementia could be difficult to activate, might not want to or simply did not have the physical strength to participate in various activities. It was important to adapt all activities to each individual.

(Enrolled nurse) *“It’s hard to get her active, she understands, she does, but she doesn’t want to. She hasn’t liked taking a short walk either. She gets furious when she goes out, she doesn’t want to, we tried when the weather was lovely, but she just doesn’t like going out. So we sit with F and talk to her and sometimes we browse through magazines we have, or read a newspaper together and talk a bit about the pictures depending on how much she’s able to and wants to.”*

Meals were among the important daily activities in the group home. They were served five or six times a day. The immigrants with dementia had lived in Sweden for many years and were accustomed to eating both Swedish and traditional food. However, when the family members described similarities in the cultures of Swedish food and the traditional food their loved one was used to, they thought that their loved one wanted more traditional food and really appreciated it when it was served.

(Daughter about E) *“Here she eats what she is given, what is included, she eats it and she likes it, when we did her shopping at home we used to buy Easter mämmi and things like that, I’ve even bought that for her here …”*

(Enrolled nurse about E) *“She doesn’t want to say that she really likes Finnish food, but she does because sometimes when she’s at her daughter’s and they cook Finnish food, she comes back and tells us what she has had to eat, and she is so happy that she’s had their dish or something Finnish, she’s very happy.”*

The family members had expectations of activities that the professional caregivers could not always meet, as they thought that activation was depending on the wishes of and the physical ability of the immigrant with dementia. On the other hand the group home was unable to satisfy the wishes for traditional food for the immigrants with dementia, which instead had to be provided by the family members.

## Challenges in communication

All the immigrants with dementia included in this study had lost their Swedish language and, except for the bilingual enrolled nurses who spoke Finnish, Hungarian or Estonian, most of the professional caregivers at the group homes were Swedish-speaking. The category ‘challenges in communication’ covers three subcategories of ‘limited communication between the immigrant with dementia and the Swedish-speaking professional caregivers’, ‘consequences of linguistic misunderstandings’ and ‘nuanced communication in a common language’.

### Limited communication between the immigrant with dementia and the Swedish-speaking nursing staff

According to the nursing staff, the most significant difference between the native Swedish PWD and the immigrant PWD was language. Having a common language in everyday life and work was important for understanding and communicating with the PWD. The Swedish-speaking caregivers tried to communicate as the best they could by using simple language and non-verbal expressions such as gestures and miming.

(Enrolled nurse) *“But her son says that she understands what I say and that you have to look at her and talk to her face to face, and then it’s much easier for her to understand Swedish. You can use body language, I use my hands and repeat things several times so that she understands what I mean. She gets across what she wants even if you can’t have a long conversation, she makes you understand what she wants. We bounce back and forth until we get it right.”*

The family member also noticed that communication was limited when the professional caregivers did not speak the native language of the immigrant with dementia, but they did not stress the importance of a common language as much as the Swedish-speaking caregivers. The family members also noticed that there were bilingual caregivers who occasionally spoke to the immigrants with dementia. Nevertheless, they noticed that not being able to communicate in a common language could have negative consequences.

### Consequences of linguistic misunderstandings

Both the family members and the professional caregivers reported that linguistic misunderstandings occurred and that one of the most serious misunderstandings was being diagnosed with dementia and ending up in the wrong type of care. One of the PWD, a Finnish-speaking lady at the group home seemed to be in the wrong kind of care. Both her family member and the nursing staff questioned her diagnosis since they did not think her symptoms were consistent with dementia.

(Enrolled nurse) *“I can’t see any memory problems. In my view, as a health professional working on a dementia ward where others have such bad dementia, I can’t see anything obvious, and she‘s reading ’Earth’s Children’, a big book. ‘Where are you in the book, I asked.’ ‘Well,’ she said ‘there are so many characters, but I have actually managed to keep track of them’ and she said how far she’d got and I checked and she was indeed correct!”*

This lady’s daughter explained that her mother had previously been heavily sedated because of other psychiatric issues. When she moved to the group home and received the correct medication, she regained the will to live. Since she moved to the group home, she had become active, started reading books and magazines and did not seem to have any major lapses in memory.

(Daughter) *“She [the mother] said ‘No, I don’t think I want to die anymore.’ and then she started getting her strength back, and she came back to me. There have only been positive changes… I’ve got her reading, I think that is important, yes, now she’s really into her books, and that’s fun and I think it’s great to hear her talk about past times, because I was so young when I came to Sweden, so I think it’s great when she starts talking and I ask her about things that happened, about people and my grandmother and relatives, and where they lived, and she enjoys talking about it, and I think it’s really interesting to listen to her, she has a super memory.”*

The daughter had asked her mother how she felt about living in the group home with other PWD and her answer was that, compared to her fellow residents, she felt very healthy and, to some extent, she tried to avoid them but liked living in the group home.

According to both the family members and the nursing staff, communication was hampered and they reported that, as a consequence of not being able to talk to the immigrants with dementia, the immigrants isolated themselves from the other residents and the nursing staff, and that their social life at the group home was concentrated to mealtimes. This social isolation was understood in different ways by the Swedish-speaking and the bilingual caregivers. The Swedish-speaking caregivers interpreted it as if the non-Swedish speaking PWD was introvert and withdrawn, the bilingual caregivers characterised the same person as being very sociable and talkative as soon as he or she was given the opportunity to speak their mother tongue.

(Swedish-speaking enrolled nurse about A) *“Well, the consequences are of course that we can’t have a conversation, and that means, uh, she becomes lonely, she becomes isolated! She must be, because she must surely think it’s difficult to talk to us as much as we think it’s difficult to talk to her and that’s just talking about the essential things we have to talk about and, how should I put it, we don’t chat socially. I was told before she moved in that she wanted to be on her own, that she’s that type of person, she wants to be alone.*

(Finnish-speaking enrolled nurse about A)*“I think she’s been very sociable, I see how happy she is when I go in to see her. ‘Are you here today?’ She tells us lots when there’s no one else there, she tells us about the little things, she tells the staff the important things, but we two chat about everything.”*

One of the Finnish-Swedish immigrants’ daughter argued that Finnish was strongly rooted in her mother, and that was why it was necessary to speak to her in her mother tongue. The daughter was also of the opinion that it was easier for her to understand her mother’s fragmented language because she felt she had more context about what her mother was saying.

(Daughter) *“… and it’s so strong, it’s so strong in her and that’s why her Finnish language is becoming more evident, though I think it’s good, because I can start speaking Finnish to her, and we’ll get more context from what is said.”*

In cases where bilingual nursing staff were available, the family member described how the bilingual nursing staff translated into the PWD’s mother tongue; however, when not available, the family members had to interpret and be spokespersons for their loved ones with dementia.

### Nuanced communication in a common language

According to the professional caregivers, the benefits of being able to speak one’s mother tongue entailed linguistic stimulation for the PWD and, depending on the deterioration in their linguistic ability, this meant that, for PWD whose language was fairly intact, they could still express themselves in a nuanced manner, enabling them to socialise and develop social relationships.

(Finnish-speaking enrolled nurse) *“I see how happy she is when I go in to see her, she has lots to tell us when nobody else is there, we talk a bit about everything, language difficulties can put a spanner in the works though, you can’t explain everything, it’s simply too difficult to explain, and it’s easier in her language.”*

Similarly, for immigrant PWD who could no longer communicate verbally, their mother tongue seemed to still be important. The nursing staff argued that they listened and reacted when they heard words in their mother tongue.

(L) *“Yes, as we say in Estonian, ‘kalli, kalli’, and she’s so happy and hugs us back.”*

## The role of the family member at the group home

The move of a loved one with dementia to a group home entailed a change in roles for the family member. From having had the main responsibility for the care of their loved ones while they lived in their own home, the family member’s role changed to being a visitor at the group home. Nevertheless, the family member’s visits were important to both the PWD and the professional caregivers. The two subcategories of ‘a link to the healthy life story of the family member with dementia’ and ‘an expert and interpreter for the nursing staff’ explains the role of the family member at the group home, comes below.

### A link to the healthy life story of the family member with dementia

For those immigrants with dementia who were married to native Swedes, the loss of Swedish language skills created a distance in their marital relationship as they were no longer able to communicate with one another. The family members of those in the final stages of the disease were also unsure as to whether their visits really mattered.

(Family member, husband) *“No, you can’t, you can’t communicate, it was already difficult when she lived up there [another nursing home], right, but to never and never, there was no context. Now there is absolutely nothing that I, I can’t understand a word myself when I visit. It’s not often [that I visit] because there’s no contact, I don’t know if she enjoys it, there’s no benefit to her from me visiting.”*

On the other hand, the professional caregivers were of a different opinion and said that visits were very important to the PWD

(Enrolled nurse) *“Yes, and he’s here a lot [the husband], of course, visiting her, and she is so happy when he comes.”*

Depending on the PWD´s medical state and level of functioning, their family member’s visits varied, and included sitting and talking to them about memories, friends and acquaintances they had in common. The family members also helped at mealtimes, they looked at magazines with their loved ones, listened to their favourite music and, on a good day, took their loved one out for a walk or a visit to the family member’s home. The family member also helped with errands and shopping for items such as traditional food for their family member with dementia.

*Daughter: “Sometimes I take her to the kiosk or to my house, or I help her do things like visiting the dentist. Then we tend to look at the newspapers, just for something to talk about, there might be something interesting, animals, she likes them, we might talk about something or read something about gardening and general stuff and we talk to each other.”*

*Husband: “I visit twice a week, I give her a banana and feed her. I think she recognises me, especially when I say, ‘servus’. When she’s eaten the banana, I say, ‘let’s talk’ and I tell her who I’ve met and visited and you can see her mouth moving, but no words come out - in the last six months I’ve asked her certain things - and she sometimes nods and I think that’s positive.”*

While the family member’s visits were socially and emotionally important to the PWD, the professional caregivers saw the family member more as a source of information about the PWD and as interpreters.

### An expert and interpreter for the nursing staff

The family knew the habits and interests of their loved ones and were able to provide information that was of help in their care. Some family members experienced that communication between the caregivers and their loved one functioned well and that they got the information they needed. The family member were always informed if there was a change in the PWD’s medical status.

(Daughter) “*The nurse in charge of this unit calls as soon as there’s something, and I think that’s very good, so I think that we have good contact with the caregivers.*”

The family member contributed information about the life story of their loved one in an admission assessment held when the PWD was admitted to the group home. Thereafter, there was continuous sharing of information between the family member and the professional caregivers regarding changes in health and other information about the PWD’s life and interests.

(Enrolled nurse) *“And we talk a lot [with the family member] and bit by bit we get some good information that’s useful to know. Then their relatives tell us their life story, and there’s an interview on arrival.”*

However, communication also seemed to depend on the kind of relationship that had developed between the professional caregivers and the family members. The caregivers were aware of the fact that the family members were not always noticed, and that the information they could provide was not always asked for. The professional caregivers meant that more could be done to develop relationships with the family members.

(Enrolled nurse) *“And we’ve talked about having little follow-ups to the arrival interviews, we’ve said that we have to get them started, they are quite important, because perhaps not everyone has the contact with their relatives that they want - when they come, maybe there’s a ‘hello’ and a few phrases exchanged and then it’s ‘goodbye’ when they go. It doesn’t feel right that we haven’t had the time to sit down and talk to them, relatives are important.”*

The family member also acted as interpreters for their loved ones with dementia and the professional caregivers.

(Daughter) *“Yes, then she doesn’t respond; she says, she asks me, even though she understands perfectly what they asked her, ‘What did she say?’ she asks in Finnish. ‘But you know really, don’t you?’”*

## Discussion

The results of this study have reaffirmed that moving to a group home was a big change for PWD. However, adjusting to their new living arrangements varied among the immigrants with dementia. Their family members were also affected by their new living situation since the main responsibility of caring for their loved one with dementia had been transferred to professional caregivers at group homes. The main concern of the family member after their loved one moved was the limited number of activities available for their loved one. This is in contrast to earlier studies that have reported that reasons for seeking help late included the family member worrying about the care environment being culturally alien to the PWD [13, 14]. However, regarding activities, it seemed that the family member had difficulties understanding the nurses’ reasoning regarding the appropriate activity levels for PWD. Nonetheless, the family members were satisfied with the general care provided at the group homes.

Communication difficulties were mainly experienced by the Swedish-speaking enrolled nurses and, in that respect, the bilingual enrolled nurses played an important role in facilitating communication, even though this was not sufficient. Previous studies show that PWD have considerable communication impairment caused by their disease [29, 30]. PWD who moved to nursing homes in the later stages of their disease experienced language problems such as difficulty in word-finding, understanding both written and spoken languages and limited vocabulary. These make communication difficult, particularly for the professional caregivers who did not know the PWD before they developed dementia symptoms. Immigrants with dementia run a significant risk of becoming socially isolated in nursing homes [31, 32]. Communication difficulties increase when they move to a group home where the language spoken is not their mother tongue [33]. This is the case for many immigrants who grow old and develop dementia in a country with a language and culture different to theirs [10]. Considering the sharp trend in aging and the fact that a large proportion of the population has an immigrant background, this issue should receive comprehensive attention from healthcare policy makers and nursing staff [34, 35]. This study revealed that enrolled nurses had difficulties communicating with PWD, which resulted in misunderstandings. These misunderstandings and the subsequent dissatisfaction for professional caregivers, PWD and their family members have a direct impact on the health and welfare of PWD and their family members. Moreover, they create ethical problems for nurses/professional caregivers [36, 37]. The results of this study are in line with other studies which indicate that the family member of PWD expect professional caregivers to facilitate access to the traditional foods and cultural activities of the PWD’s country of origin [10, 38] regardless of how long they have lived in their current location. Even though the immigrants with dementia in our study had moved to Sweden at least 60 years earlier, their family members perceived traditional food as being important. This should be noted by professional caregivers and healthcare planners considering the growing diversity in today’s society in terms of ethnicity and cultural background.

The family member in this study played a crucial role in the care and quality of everyday life of immigrant PWD in the group homes by facilitating communication between the nursing staff and PWD and by also providing possibilities for PWD to access desired cultural activities which the nursing staff did not recognise were needed or could not deliver. In view of society’s limited resources regarding bilingual staff and nursing homes designed for immigrants with dementia, the results of this study show that involving the immigrants’ family member is of great value. Considering what transpired when the family members visited, by bringing in things familiar to the PWD or talking about memories, events and people they had in common, the PWD could, perhaps, regain contact with their lost life and retain a fuller sense of their identity. The visits of family members are certainly important for all PWD but perhaps even more so for an immigrant with dementia, since their family member (usually) speaks the same language and they can recall memories in a way that is not possible in a foreign language caregiving environment. However, being able to talk to immigrants with dementia in their language is not always enough. Ekman [39] showed that even when PWD were able to talk to Swedish professional caregivers, they had communication problems. Language is more than just communication. It is a reflection of a view of the world [40] the person has lived in. Therefore, not only people who are able to communicate in a certain language but people from that particular cultural background are needed to assist professional caregivers in meeting PWD in their world. The results here show that the family member’s understanding of housing, everyday life and the need for activities was different to that of nurses, and both sides had difficulties in comprehending each other. According to a study by Holmgren, Emami, Eriksson & Eriksson [41] a barrier to involving the family member in actual caregiving might be the professional caregivers’ perception of their professional role and their limited understanding of the family member’s importance to the wellbeing of older people in residential care. If the family member is the only link to the life lost to the immigrant with dementia, professional caregivers need to rethink the involvement of family members and start perceiving them as PWD experts. This shift in perception regarding the involvement of family member in residential care could contribute to a more fulfilling and holistic dimension for PWD. More research is needed to shed light on the ways family members can contribute to promoting the health and wellbeing of immigrants with dementia in group homes. Professional caregivers also need to be informed of the importance of family member’s involvement in care relationships and practices as well as strategies for an effective collaboration that benefits both the PWD and their family members.

### Methodological considerations

This study is exploratory in the sense that immigrants with dementia living in Swedish group homes comprise a relatively unexplored group; therefore, a qualitative approach was considered suitable. The people in this study have been studied in a caregiving context, which is common when PWD have moderate to severe dementia and need constant care in a nursing home. Since it is common for immigrants with dementia to receive care in Swedish-speaking group homes, it was considered appropriate to study such a caregiving context.

The decision to include both family members and caregiving personnel was taken in order to obtain a variety of experiences. In addition, it was considered possible to study the family members’ and the professional caregivers’ perspectives at the same time. By including the perspectives of both the family member and nursing staff, that which was said by one group could be verified by the other, as they both talked about the same phenomena independently. One of the study’s limitations is its small sample size. However, as the study was explorative in nature, a smaller number of participants was considered sufficient to answer the research question. It is possible that the picture of the care provided in the group home would have been richer if interviews with the PWD had been included. Nonetheless, as the aim of this study was to explore the experiences of the family member and their professional caregivers, their perspectives were in focus. As it turned out, three bilingual professional caregivers could also contribute with additional valuable information; consequently, they were also included.

The transcribed interviews were analysed by the first (SPR) and the second authors (MS). The categories were discussed with the third author (MM) until consensus was achieved. To ensure trustworthiness, the whole study has also been described as clearly as possible.

The authors’ (SPR, MS, MM) levels of prejudice and own immigrant backgrounds may have influenced their interpretation of the results; however, by discussing together, a professional distance has been maintained. In addition, the first author (SPR) kept journal notes about the roles of the researchers during the data collection.

## Conclusions

The family member of immigrants with dementia play a crucial role in the lives of immigrant PWD in group homes. They facilitate communication between professional caregivers and people with dementia and also provide possibilities for immigrants with dementia to access desired cultural activities that professional caregivers do not recognise as needed or cannot deliver. Importance of family members of immigrants with dementia get underlined to the possibilities they provide for people with dementia in mending their broken access to their earlier life and identities. More research is needed to explore ways family members can contribute in promoting the health and wellbeing of PWD in group homes.

## Abbreviation

PWD: Means people or person/persons with dementia including all ethnic backgrounds
